# A High Triglyceride-Glucose Index Value Is Associated With an Increased Risk of Carotid Plaque Burden in Subjects With Prediabetes and New-Onset Type 2 Diabetes: A Real-World Study

**DOI:** 10.3389/fcvm.2022.832491

**Published:** 2022-03-03

**Authors:** Zhen-zhen Jiang, Jian-bo Zhu, Hua-liang Shen, Shan-shan Zhao, Yun-yi Tang, Shao-qi Tang, Xia-tian Liu, Tian-an Jiang

**Affiliations:** ^1^Department of Ultrasound Medicine, The First Affiliated Hospital, Zhejiang University School of Medicine, Hangzhou, China; ^2^Department of Ultrasound, Shaoxing People's Hospital (Shaoxing Hospital, Zhejiang University School of Medicine), Shaoxing, China; ^3^Key Laboratory of Pulsed Power Translational Medicine of Zhejiang Province, Hangzhou, China; ^4^Zhejiang University Cancer Center, Zhejiang, China

**Keywords:** triglyceride-glucose index, atherosclerosis, carotid plaque, prediabetes, new-onset type 2 diabetes

## Abstract

**Background:**

The triglyceride-glucose (TyG) index has been proposed as a convincing indicator of insulin resistance and has been found to be associated with atherosclerosis among diabetic patients. However, the relationship between the TyG index and arteriosclerosis in subjects with prediabetes and new-onset type 2 diabetes (T2D) remains uncertain. The purpose of this study was to assess the degree of carotid plaque burden in patients with prediabetes and new-onset T2D and to investigate the association between the TyG index and the degree of carotid plaque burden in this population.

**Methods:**

This was a cross-sectional observational study that included 716 subjects aged 40–70 years old with prediabetes or new-onset T2D. Demographic, anthropometric, and laboratory measurements were collected. Participants underwent carotid arteriosclerosis evaluation by ultrasonography, and the degree of atherosclerosis was evaluated according to the carotid plaque burden. The TyG index was calculated.

**Results:**

The population was stratified into high or low TyG index groups according to the median TyG index value. Higher values were associated with a higher BMI and waist circumference as well as higher total cholesterol, triglyceride, low-density lipoprotein cholesterol, plasma glucose, glycated hemoglobin, fasting C-peptide, and C-reactive protein levels (*P* < 0.001). The high TyG index group had a higher atherosclerotic plaque burden than the low TyG index group (*P* < 0.001). Multiclassification logistic regression analysis showed that the TyG index was positively associated with a high plaque burden [odds ratio (OR): 16.706, 95% confidence interval (CI): 3.988–69.978, *P* = 0.000], while no association was found between the TyG index and a low/moderate plaque burden. This association remained consistent in the subgroup analysis. In multiple linear regression analysis, sex, age, and the TyG index were found to be independently associated with carotid plaque burden. For each unit increase in the TyG index, the risk of a high carotid plaque burden increased 1.595-fold.

**Conclusion:**

A high TyG index was positively associated with a high carotid plaque burden in subjects with prediabetes and new-onset T2D. Clinicians should pay close attention to the TyG index to help these patients receive the greatest benefit from early intervention.

## Introduction

Diabetes mellitus, a leading cause of disability worldwide, is a well-known risk factor for atherosclerotic cardiovascular disease (ASCVD) (1). A previous study indicated that early identification of diabetic individuals at high risk for cardiovascular events should be of a clinical priority because timely prevention and subsequent rapid interventions may reduce the disability and mortality rates in these patients (2).

Indeed, in the prediabetic state, the risk of cardiovascular events is persistent, and atherosclerosis might appear prior to a diagnosis of diabetes mellitus (3, 4). According to previous studies, subjects with prediabetes, and new-onset type 2 diabetes (T2D) may represent a specific population with increased cardiovascular risk, and thus, different prevention programs should be applied (5). Therefore, assessment of the progression of atherosclerosis in patients with prediabetes and new-onset T2D is critical for optimizing and personalizing treatment strategies.

Carotid plaque burden plays an essential role in the progression of ASCVD. For the early diagnosis of arteriosclerosis, imaging modalities such as ultrasound and coronary angiography are usually needed, and performing such procedures during routine monitoring of an asymptomatic population on a large scale may be difficult as they are expensive and time-consuming. Therefore, early recognition of simple and accurate indicators of carotid plaque burden that can be applied in daily clinical practice could aid in the early identification of patients at high risk of ASCVDs (6). Insulin resistance (IR) has been confirmed to be generally present before the onset of T2D and plays a major role in the development of atherosclerosis (7, 8). Triglyceride-glucose (TyG) index, which has been proposed as a convincing indicator of IR (9), has been demonstrated to be associated with the prevalence of ASCVD (10, 11). However, the study populations have mainly consisted of patients with diabetes (12–14). To the best of our knowledge, there is little evidence on the correlation between the TyG index and carotid plaque burden in patients with prediabetes and new-onset T2D to date.

Based on the above background, the current study evaluated whether and how the simple calculated TyG index was associated with the carotid plaque burden in subjects with prediabetes and new-onset T2D without any ASCVDs. The results of this work can contribute to the early recognition of patients at high risk of cerebrovascular accidents.

## Materials and Methods

### Study Design and Subjects

Between January 2018 and January 2021, a total of 4,394 hospitalized patients with abnormal blood glucose were screened. After a review of clinical information consisting of medication usage data, self-reported medical history, glycated hemoglobin (HbA1c), fasting blood glucose, and oral glucose tolerance test (OGTT) results, 992 patients with a diagnosis of prediabetes or new-onset T2D at our hospital were included. The inclusion criteria were as follows: (1) diagnosis of prediabetes or new-onset T2D; (2) age between 40 and 70 years old; (3) no history of atherosclerotic cardiovascular disease; (4) no severe renal dysfunction; (5) no previous lipid-lowering treatment; and (6) carotid ultrasonography.

The criteria for prediabetes included a fasting plasma glucose (FPG) level between 100 mg/dL (5.6 mmol/l) and 125 mg/dL (6.9 mmol/l) and/or an HbA1c ranging between 5.7 and 6.4%, without a history of diabetes or the use of any antidiabetic drugs. Criteria for new-onset T2D included an incidental finding of FPG ≥ 126 mg/dL (7.0 mmol/l) and/or HbA1c ≥ 6.5% for no more than 6 months, without a history of diabetes or the use of any antidiabetic drugs (15, 16). Atherosclerotic cardiovascular disease included ischemic stroke, transient ischemic attack, coronary artery disease, heart failure, and arteriosclerosis obliterans. Severe renal dysfunction was defined as an estimated glomerular filtration rate (eGFR) <30 mL/min.

**Figure 1:**
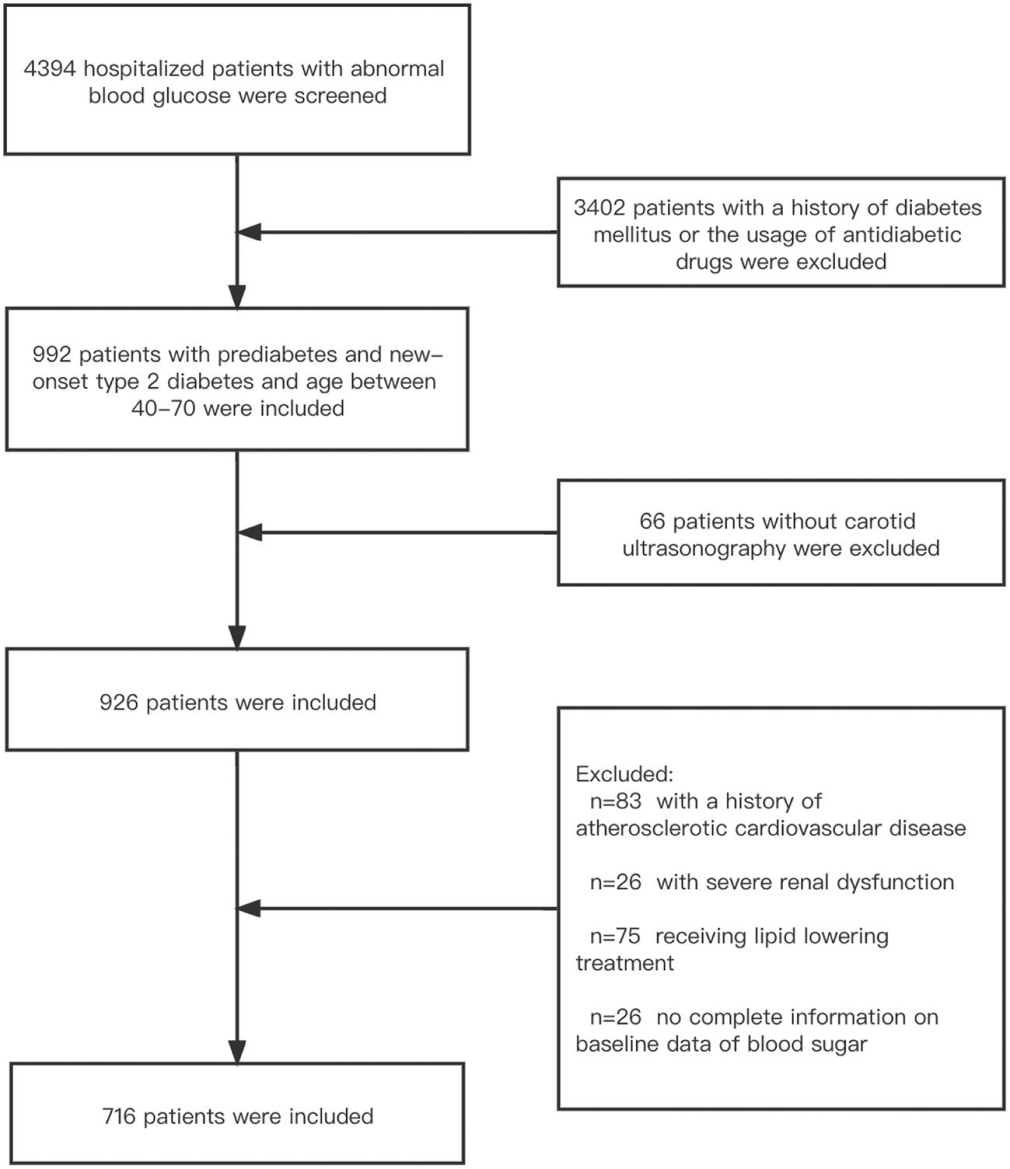
Enrollment flowchart of the study population.

Based on the inclusion criteria, 276 patients were excluded, and 716 patients were ultimately included in the analysis (Figure 1). The study was conducted according to the principles of the Declaration of Helsinki and approved by the Ethics Committee of Shaoxing Hospital, Zhejiang University School of Medicine. The data were anonymous, and the requirement for informed consent was therefore waived.

### Data Collection and Laboratory Measurements

Demographic information (including sex and age) and anthropometric measures (including height, weight, and waist circumference) were collected. Body mass index (BMI) was calculated using the equation weight (kg)/squared value of height (m^2^).

Clinical data such as tobacco use, alcohol use and history of hypertension were also recorded. Hypertension was defined as systolic blood pressure (SBP) ≥ 140 mmHg or diastolic blood pressure ≥ 90 mmHg on two different occasions or a history of antihypertensive therapy.

Venous blood samples were collected from the subjects after an overnight fasting for the analysis of total cholesterol (TC), triglycerides (TGs), high-density lipoprotein cholesterol (HDL-C), low-density lipoprotein cholesterol (LDL-C), FPG, HbA1c, and high-sensitivity C-reactive protein (hs-CRP). We calculated the TyG index using the equation log [(fasting TG (mg/dl) × FPG (mg/dl)/2] (17).

### Assessment of Carotid Plaques

Carotid ultrasonography was performed by trained and experienced sonographers using a color ultrasound diagnostic apparatus equipped with a linear array transducer. The common, bifurcation, external, and internal carotid arteries were examined bilaterally from the transverse and longitudinal orientations to evaluate the carotid intima-media thickness (IMT), the presence and morphology of atherosclerotic plaque, and the presence of carotid stenosis.

Carotid intimal thickening was defined as 1.0 ≤ IMT ≤ 1.5 mm, and atherosclerotic plaque was defined as a localized IMT ≥ 1.5 mm or a relative focal thickening of more than 50% of the IMT of the surrounding tissue. A finding of two or more plaques was defined as multiple plaques. According to morphology and echo characteristics, we divided the plaques into stable plaques or unstable plaques. Plaques with hyperechoic, homogeneous echoes and a smooth surface were defined as stable plaques. Hypoechoics plaques and those with mixed echoes or ulcerations were defined as unstable plaques (18). Carotid stenosis was defined as any degree of narrowing resulting from plaques. The carotid plaque burden of each subject was evaluated using a modified plaque scoring system (19). Briefly, for each subject, the unilateral carotid artery was divided into four regions: the common carotid artery region, carotid bulb and bifurcating region, internal carotid region, and external carotid region. Each region received a score from 1 to 3, with 1 being a single plaque, 2 being multiple plaques, and 3 being stenosis. A total score was obtained by adding the scores of the eight regions together, and then the carotid plaque burden could be assessed. The plaque score (PS) was classified into PS_1_: low plaque burden (0 < PS <3, single plaque in no more than three regions or multiple plaques in no more than two regions); PS_2_: moderate plaque burden (3 ≤ PS ≤ 6, multiple plaques in no more than three regions or carotid stenosis in no more than two regions); and PS_3_: high plaque burden (PS > 6, multiple plaques in more than three regions or carotid stenosis in more than two regions). The carotid ultrasonography results were reviewed by two independent experienced sonographers blinded to the clinical data. Discrepancies were resolved by consensus.

### Statistical Analysis

The subjects were divided into two groups based on their TyG index values (high or low), and the baseline clinical data and ultrasound findings of the two groups were compared. The Kolmogorov–Smirnov test was used to evaluate the normality of the distribution of continuous variables. Data are presented as medians (interquartile ranges) for continuous variables with a non-normal distribution and as percentages for categorical variables. Group differences were analyzed using the Wilcoxon rank sum test for continuous variables and the chi-square test for categorical variables. A chi-square test for trend was used to assess the trend of carotid plaque burden prevalence with a high TyG index. To assess the possible influencing factors of carotid plaque burden of the subjects, multiclassification logistic regression analysis was performed by calculating odds ratios (ORs) and 95% confidence intervals (CIs). Finally, we developed a linear regression model to explore the correlation between the change in the TyG index and the carotid plaque burden after adjusting for sex, age, tobacco use, TGs, HDL-C, FPG, and HbA1c. A *P* ≤ 0.05 was considered indicative of statistical significance. All statistical analyses were performed by using SPSS version 21.0 (SPSS Inc., Chicago, IL).

## Results

### Characteristics of the Subjects

The demographic and baseline data are presented in Table 1. The median age of the subjects was 55 years (interquartile range: 49–61 years), and 469 (65.50%) subjects were male. A total of 559 (78.07%) subjects had new-onset T2D. Regarding arteriosclerosis risk factors among the participants, 46.93% had hypertension, 43.16% were current smokers, and 37.29% currently consumed alcohol.

**Table 1:** Baseline characteristics of the subjects stratified according to TyG index.

	**Overall (*n* = 716)**	**High TyG index group (*n* = 358)**	**Low TyG index group (*n* = 358)**	***P*-value**
Age (years)	55 (49–61)	54 (48–60)	56 (50–62)	0.002*
Men (*n*, %)	469 (65.50)	245 (68.44)	224 (62.57)	0.099
New-onset T2D (*n*, %)	559 (78.07)	335 (93.58)	224 (62.57)	<0.001^*^
BMI (kg/m^2^)	24.70 (22.40–26.90)	25.30 (22.95–27.40)	24.29 (22.05–26.43)	<0.001^*^
Waist circumference (cm)	88.00 (82.00–94.00)	90.00 (84.00–95.88)	85.00 (80.00–92.75)	<0.001^*^
SBP (mmHg)	131.00 (119.25–144.00)	130.00 (119.00–143.00)	132.00 (119.75–145.00)	0.655
DBP (mmHg)	83.00 (75.00–90.00)	84.50 (77.00–90.00)	81.00 (74.00–89.00)	0.003^*^
Hypertension (*n*, %)	336 (46.93)	162 (45.25)	174 (48.60)	0.369
Current smoking (*n*, %)	309 (43.16)	163 (45.53)	146 (40.78)	0.200
Alcohol consumption (*n*, %)	267 (37.29)	134 (37.43)	133 (37.15)	0.938
Total cholesterol (mmol/L)	4.72 (4.01–5.52)	5.21 (4.38–6.10)	4.31 (3.69–4.93)	<0.001^*^
Triglyceride (mmol/L)	1.51 (1.12–2.25)	2.20 (1.68–2.97)	1.14 (0.94–1.44)	<0.001^*^
HDL cholesterol (mmol/L)	1.08 (0.92–1.30)	1.05 (0.91–1.24)	1.13 (0.97–1.34)	0.001^*^
LDL cholesterol (mmol/L)	3.06 (2.47–3.69)	3.44 (2.74–4.01)	2.78 (2.28–3.25)	<0.001^*^
Hs-CRP (mg/L)	1.56 (0.76–3.31)	1.88 (1.02–3.47)	1.26 (0.55–3.21)	<0.001^*^
Fasting blood glucose (mmol/L)	10.20 (7.34–13.22)	12.09 (9.60–14.52)	8.12 (6.21–10.66)	<0.001^*^
TyG index	7.84 (7.39–8.28)	8.28 (8.05–8.62)	7.39 (7.11–7.61)	<0.001^*^
Fasting C-peptide (pmol/L)	489.31 (345.41–646.16)	512.00 (369.10–666.41)	470.00(333.52–617.87)	0.047^*^
Fasting insulin (pmol/L)	35.01 (24.40–52.40)	36.41 (24.99–53.30)	34.00(23.93–51.02)	0.159
HbA1c (%)	10.30 (8.20–12.10)	10.80 (9.30–12.50)	9.50 (7.05–11.70)	<0.001^*^

*Data are presented as number (%) or median (P_25_-P_75_). TyG, triglyceride-glucose; T2D, type 2 diabetes; BMI, body mass index; SBP, systolic blood pressure; DBP, diastolic blood pressure; HDL, high density lipoprotein; LDL, low density lipoprotein; hs-CRP, high sensitivity C-reactive protein; HbA1c, hemoglobin.*

* *indicates data with statistical significance (P ≤ 0.05)*.

The median value of the TyG index (*M* = 7.84) was used to divide the participants into high and low TyG index groups, and subgroup analyses of the data were performed. Subjects with a higher TyG index value were more likely to have higher BMI and waist circumference (25.30 [22.95–27.40] vs. 24.29 [22.05–26.43)] kg/m^2^, *P* < 0.001 and 90.00 [84.00–95.88] vs. 85.00 [80.00–92.75] cm, *P* < 0.001, respectively) than subjects with a lower TyG index value. TC and TG values were higher in the high TyG index group than in the low TyG index group (5.21 [4.38–6.10] vs. 4.31 [3.69–4.93)] mmol/L, *P* < 0.001 and 2.20 [1.68–2.97] vs. 1.14 [0.94–1.44] mmol/L, *P* < 0.001, respectively). Accordingly, the high TyG index group exhibited a higher level of LDL-C (3.44 [2.74–4.01] vs. 2.78 [2.28–3.25)] mmol/L, *P* < 0.001) and a lower level of HDL-C (1.05 [0.91–1.24] vs. 1.13 [0.97–1.34] mmol/L, *P* = 0.001) than the low TyG index group. Moreover, FPG, HbA1c, and fasting C-peptide levels were higher in the high TyG index group than in the low TyG index group (12.09 [9.60–14.52] vs. 8.12 [6.21–10.66] mmol/L, *P* < 0.001, 10.80 [9.30–12.50] vs. 9.50 [7.05–11.70] %, *P* < 0.001, and 512.00 [369.10–666.41] vs. 470.00 [333.52–617.87] pmol/L, *P* < 0.05, respectively). Finally, subjects in the high TyG index group had higher levels of hs-CRP than those in the low TyG index group (1.88 [1.02–3.47] vs. 1.26 [0.55–3.21] mg/L, *P* < 0.001).

### Degree of Carotid Arteriosclerosis of the Subjects

**Figure 2:**
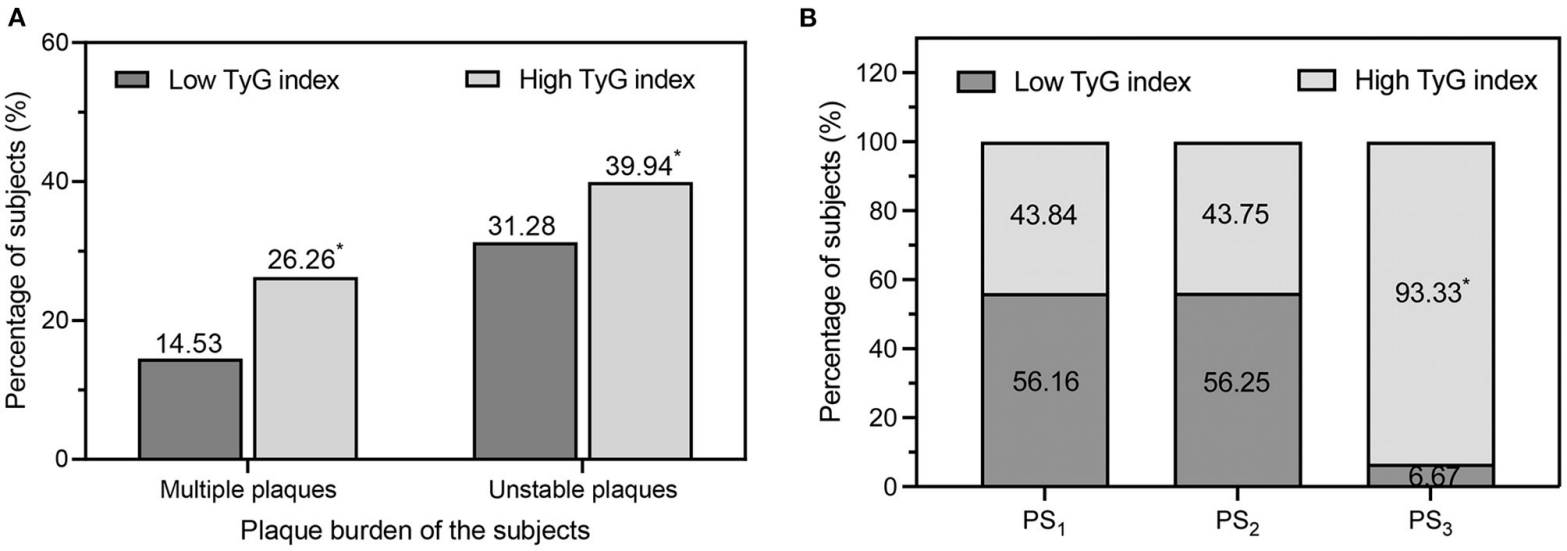
**(A)** Comparison of the percentage of multiple plaques and unstable plaques in the low TyG index group and high TyG index group. **(B)** Percentages of low/high TyG index values in different categories of carotid plaque burden degree. PS_1_, low plaque burden; PS_2_, moderate plaque burden; PS_3_, high plaque burden. PS, plaque score; TyG, triglyceride-glucose. *P < 0.01 vs. low TyG index.

The degree of carotid arteriosclerosis for the subjects is listed in Table 2. The present study found that there was no difference in carotid IMT measurements or the prevalence of IMT thickening and plaques between the high and low TyG index groups. However, the high TyG index group had a higher atherosclerotic plaque burden than the low TyG index group, with more patients with multiple plaques (26.26 vs. 14.53%, *P* < 0.001), more unstable plaques (39.94 vs. 31.28%, *P* < 0.001), and a higher PS (2.00 [1.00–6.00] vs. 2.00 [1.00–4.00], *P* < 0.001; Figure 2).

**Table 2:** Degree of carotid arteriosclerosis of the subjects.

**Degree of carotid arteriosclerosis**	**High TyG index group (*n* = 358)**	**Low TyG index group (*n* = 358)**	***P*-value**
CIMT (mm)	0.80 (0.70–0.90)	0.80 (0.70–0.90)	0.693
CIMT thickening (*n*, %)			0.150
No	313 (87.43)	325 (90.78)	
Yes	45 (12.57)	33 (9.22)	
Carotid plaque (*n*, %)			0.881
No	185 (51.68)	187 (52.23)	
Yes	173 (48.32)	171 (47.77)	
Number of plaques, *n* (%)			<0.001^*^
=0	185 (51.68)	187 (52.23)	
=1	79 (22.07)	119 (33.24)	
≥2	94 (26.26)	52 (14.53)	
Plaque stability (*n*, %)			0.001^*^
No plaque	185 (51.68)	187 (52.23)	
Stable plaque	30 (8.38)	59 (16.48)	
Unstable plaque	143 (39.94)	112 (31.28)	
Plaque thickness (mm)	2.10 (1.80–2.70)	2.10 (1.60–2.50)	0.219
Plaque score	2.00 (1.00–6.00)	2.00 (1.00–4.00)	<0.001^*^
Number of PS, n (%)			0.004^*^
PS_1_	96 (96/219, 43.84)	123 (123/219, 56.16)	
PS_2_	35 (35/80, 43.75)	45 (45/80, 56.25)	
PS_3_	42 (42/45, 93.33)	3 (3/45, 6.67)	

*Data are presented as number (%) or median (P_25_-P_75_). CIMT, carotid intima-media thickness; TyG, triglyceride-glucose; PS_1_, low plaque burden; PS_2_, moderate plaque burden; PS_3_, high plaque burden.*

* *indicates data with statistical significance (P ≤ 0.05)*.

With an increasing PS, the proportion of subjects with a high TyG index increased significantly, reaching 93.33% in the PS_3_ category (*P* for trend =0.004). On the other hand, the proportions of subjects with a high TyG index were similar between PS_1_ and PS_2_ (43.84 vs. 43.75%, *P* > 0.001, Figure 2).

### Association of the TyG Index With Carotid Plaque Burden

Univariate logistic regression analysis was conducted with the possible influencing factors of carotid plaque burden as independent variables and PS as the dependent variable. Factors that were statistically significant in the univariate analysis were introduced into the unordered multiclassification logistic regression model, and the analysis results are shown in Table 3. Compared with subjects without a carotid plaque burden, those with a low plaque burden were more likely to be older, and the OR was 1.763 (95% CI, 1.238–2.511). Those with a moderate plaque burden were more likely to be older and current smokers and had a history of hypertension, with ORs of 4.960 (95% CI, 2.728–9.018), 1.896 (95% CI, 1.000–3.594), and 2.155 (95% CI, 1.267–3.666), respectively. Those with a high plaque burden were more likely to be older, had a history of hypertension, and had a high TyG index, with ORs of 3.183 (95% CI, 1.572–6.444), 2.477 (95% CI, 1.236–4.965), and 16.706 (95% CI, 3.988–69.978), respectively. The TyG index was positively associated with a high plaque burden (*P* =0.000), while no association between the TyG index and a low/moderate plaque burden was found in the study population.

**Table 3:** Multiclassification logistic regression analysis.

	**Carotid plaque burden**								
	**PS_1_**			**PS_2_**			**PS_3_**		
	**OR**	**95% CI**	***P***	**OR**	**95% CI**	***P***	**OR**	**95% CI**	***P***
Age (Ref <55)	1.763	1.238–2.511	0.002^*^	4.960	2.728–9.018	0.000^*^	3.183	1.572–6.444	0.001^*^
Smoking (Ref no)	1.073	0.696–1.654	0.749	1.896	1.000–3.594	0.050^*^	1.900	0.844–4.276	0.121
Hypertension (Ref no)	1.402	0.986–1.996	0.060	2.155	1.267–3.666	0.005^*^	2.477	1.236–4.965	0.011^*^
TyG index (Ref <7.84)	0.899	0.534–1.513	0.689	0.782	0.361–1.693	0.536	16.706	3.988–69.978	0.000^*^

*Multiclassification logistic regression model was used to estimate Odds ratio (OR) and 95% confidence interval (CI). Dependent variable: carotid plaque burden. PS_1_, low plaque burden; PS_2_, moderate plaque burden; PS_3_, high plaque burden. Model was adjusted for sex, age, smoking, hypertension, triglyceride, and fasting blood glucose. PS, plaque score; Ref, reference; TyG, triglyceride-glucose.*

* *indicates data with statistical significance (P ≤ 0.05)*.

**Figure 3:**
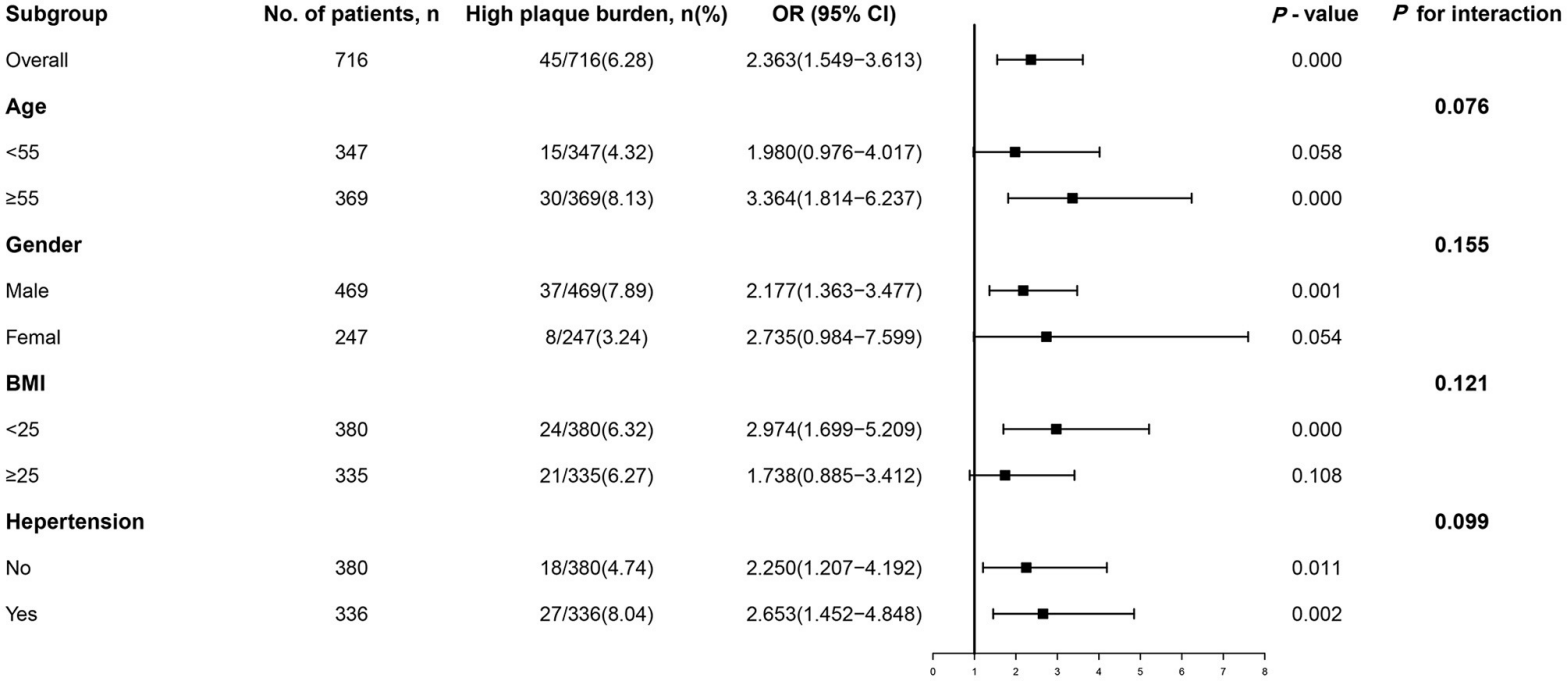
Subgroup analysis of the association between the TyG index and high plaque burden. Logistic regression after adjustment for triglycerides and fasting blood glucose was performed in subgroups according to age, sex, BMI, hypertension, smoking, and alcohol consumption. OR, odds ratio; CI, confidence interval; BMI, body mass index; TyG, triglyceride-glucose.

Even when subjects were stratified by age (<55 or ≥55 years), sex (male or female), BMI (<25 or ≥25 kg/m^2^), and hypertension (no or yes), the association between the TyG index and incident high plaque burden remained consistent (all *P* for interactions > 0.05, Figure 3). The ORs of incident high plaque burden tended to be higher in populations who were elderly, female, lean, or hypertensive than in those who were younger, male, obese, or non-hypertensive (OR [95% CI] = 3.364 [1.814–6.237], 2.735 [0.984–7.599], 2.974 [1.699–5.209], and 2.653 [1.452–4.848], respectively, *P* ≤ 0.05; Figure 3).

To further evaluate the extent to which the TyG index was associated with carotid plaque burden in our population, multiple linear regression analysis was performed. First, possible risk or protective parameters were included in the univariate analysis, and the results showed that sex, age, smoking, triglycerides, HDL cholesterol, fasting blood glucose, HbA1c, and the TyG index were meaningful. These parameters were then used to construct a regression model by using a multiple linear regression equation, and the results are displayed in Table 4. Sex, age, and the TyG index had a positive correlation with the carotid plaque burden (*b* = 0.848, *t* = 2.139, *P* = 0.033; *b* = 0.077, *t* = 4.000, *P* < 0.001; *b* = 1.595, *t* = 2.748, *P* = 0.006, respectively). TyG index showed the strongest association with the carotid plaque burden. For each unit increase in the TyG index value during this period, the plaque burden score increased 1.595-fold.

**Table 4:** Multiple linear regression analysis.

	**B**	**SE**	**T**	**95% CI**	***P-value***
Sex	0.848	0.397	2.139	0.068 to 1.628	0.033^*^
Age	0.077	0.019	4.000	0.039 to 0.116	0.000^*^
Smoking	0.258	0.359	0.720	−0.448 to 0.964	0.472
Triglyceride (mmol/L)	−0.045	0.243	−0.184	−0.524 to 0.434	0.854
HDL cholesterol (mmol/L)	−0.416	0.490	−0.848	−1.380 to 0.549	0.397
Fasting blood glucose (mmol/L)	−0.606	0.072	−0.834	−0.201 to 0.081	0.405
HbA1c (%)	0.015	0.078	0.194	−0.138 to 0.168	0.847
TyG index	1.595	0.580	2.748	0.453 to 2.737	0.006^*^

*Dependent variable: carotid plaque burden. B, linear regression coefficient; SE, standard error; CI, confidence interval; Ref, reference; HDL, high density lipoprotein; TyG, triglyceride-glucose.*

* *indicates data with statistical significance (P ≤ 0.05)*.

## Discussion

This cross-sectional study shows for the first time that a high TyG index value in subjects with prediabetes and new-onset T2D is significantly associated with an increased risk of high carotid plaque burden as assessed by ultrasound. The association remained consistent even when the subjects were stratified by age, sex, BMI, and hypertension. No association between the TyG index and a low/moderate plaque burden was found. Furthermore, we found that compared with other factors, the TyG index showed the strongest association with the carotid plaque burden. For each unit increase in the TyG index, the risk of a high carotid plaque burden increased 1.595-fold.

Prediabetes is an intermediate stage in which glucose tolerance progresses from normal to uncontrolled intolerance. As a multifactorial metabolic disorder, prediabetes has been confirmed to have a causal relationship with ASCVD (20, 21). In addition, new-onset diabetes negatively affects the morbidity and mortality rates of ASCVD to the same extent as diabetes (22). Studies have shown that arteriosclerosis is more pronounced in patients with prediabetes and new-onset diabetes than in patients with normal glucose tolerance (23). Therefore, early personalized management of subjects with prediabetes and new-onset diabetes is an important issue. However, few studies about the arteriosclerosis in these populations are available, which may hinder the identification of who should be fast-tracked into a prevention programmer during the early stage of diabetes (24). In the present study, 157 prediabetes patients and 559 new-onset diabetes inpatients were selected as research subjects for the evaluation of possible biological indicators for the detection of arteriosclerosis. Most of these people found abnormal blood glucose accidentally in the health check-up or other physical discomfort. Before the diagnosis of prediabetes or new-onset diabetes was made, none of them had ever taken any antidiabetic treatments. We united prediabetes and new-onset T2D in one group because they may represent the early stage of diabetes. The recognition of an economical and practical marker for the plaque burden in this population could help these patients receive the greatest benefit from early intervention.

In previous studies, one of the difficulties in identifying the risk factors involved in arteriosclerosis progression was the quantification of arteriosclerotic severity. In the present study, carotid plaque burden was used as a marker of atherosclerosis, which manifested as the change in the plaque score obtained from different carotid districts. As reported in the literature, the prevalence of carotid artery plaque shows a good correlation with the presence of atherosclerosis and can well reflect the overall severity of atherosclerosis in the vasculature (25, 26). The association between the TyG index and arteriosclerosis has been demonstrated in some previous studies. Guo et al. reported that the TyG index was independently associated with the arterial stiffness of peripheral arteries evaluated by brachial-ankle pulse wave velocity (27). Research has also shown that the TyG index, as a surrogate for IR, is a marker of the presence of non-calcified or mixed plaques in coronary arteries in asymptomatic individuals (28). Other studies support that a higher TyG index value is associated with the severity of coronary artery stenosis in not only patients with acute ST-elevated myocardial infarction but also asymptomatic patients with T2D (26, 29). However, their focus of these studies falls outside the areas of carotid arteries. The results from a Spanish study that included 191 participants showed that IR assessed by the TyG index was independently associated with preclinical carotid atherosclerosis in type 1 diabetes subjects (30). In addition, the TG-to-HDL ratio, another novel and simple indicator of IR, can evaluate atherosclerotic extension in prediabetic subjects and may be useful for identifying subjects at high cardiovascular risk. Nevertheless, this study did not further investigate the possible association between IR and atherosclerosis extension (31). In addition, the aforementioned studies did not evaluate the role of the TyG index in assessing the carotid plaque burden among subjects with prediabetes and new-onset T2D. In our population, the differences in carotid IMT measurements and the prevalence of IMT thickening, which are subclinical indicators of arteriosclerosis (32), were no significant between the high and low TyG index groups. Also, the percentages of subjects suffering carotid plaque between high and low TyG index group were similar. However, in the population with carotid plaques, more patients with multiple plaques and more unstable plaques were found in the high TyG index group than in the low TyG index group, indicating that the TyG index may be a more specific indicator of arteriosclerotic plaque burden in subjects with prediabetes and new-onset T2D.

In the present study, we observed that advanced age is a risk factor for different degrees of carotid arteriosclerosis. Compared with no plaque burden, the risk was increased by 1.763 times in the low plaque burden group, 4.960 times in the moderate plaque burden group, and 3.183 times in the high plaque burden group, which was consistent with previous reports, emphasizing the importance of age as a clinical risk factor for ASCVD events (33). Considering smoking as a risk factor, the association between smoking and plaque burden was not substantial, with only a weak correlation observed between smoking and moderate plaque burden in the carotid artery (*P* = 0.05). This could be explained by the possibility that plaque erosion, but not plaque burden, is the intermediate between smoking and ASCVD (34). Hypertension was found to be correlated with moderate and high plaque burden, with the risks of prevalence increasing to 2.155-fold for patients with a moderate plaque burden and 2.477-fold for those with a high plaque burden. Hypertension plays a significant role in the development of atherosclerosis. For those with uncontrolled hypertension, standard risk management programs have been effective (35), which implies that early consideration of hypertension-induced systematic chronic damage earlier could improve the current standard clinical management. The TyG index was positively associated with a high plaque burden, and no association was found with a low/moderate plaque burden in our study population. In the subjects with a high TyG index value, the prevalence of a high plaque burden was increased between 3.988- and 69.978-fold compared with that of no plaque burden, indicating that this index may be a specific indicator of a high plaque burden in asymptomatic subjects with prediabetes and new-onset T2D. We subsequently analyzed the extent to which the TyG index was associated with carotid arteriosclerosis. For each one-unit increase in the TyG index, the plaque burden score increased by 1.595-fold, which further confirmed that the TyG index may serve as a low-cost and simple indicator in evaluating the prevalence of carotid plaque burden for patients with prediabetes and new-onset T2D.

In our subgroup analysis, we found that the positive relationship between the TyG index and high plaque burden was consistent across all subgroup variables and seemed to be more evident in individuals who were elderly, female, lean or hypertensive. Age, sex, BMI, and blood pressure are well-known traditional risk factors that affecting arteriosclerosis. Interestingly, in our BMI stratified subgroup, non-obese subjects showed a higher risk of TyG-related plaque burden than obese subjects. Similar results were found in some other studies. An investigation conducted in 4,285 Korean non-obese adults over 12 years showed that a higher TyG index value significantly predicted T2D among community-dwelling lean people (36). In addition, Zhang et al. revealed that even for non-obese individuals, the TyG index can predict the risk of diabetes (37). Unlike the prevalence of obesity-related diabetes in Western countries, the proportion of non-obese subjects with diabetes is higher in Asian populations, which may be due to factors related to ethnic and lifestyle differences (36). Insulin sensitivity may also be affected by adipose tissue. The lack of subcutaneous fat makes lean people more susceptible to hypertriglyceridemia, which may lead to dysfunctional β-cell dysfunction and IR (38). As a result, lean individuals may be more sensitive to an increased TyG index value (39).

Although the potential mechanism of the relationship between the TyG index and carotid plaque burden is unclear, it may be relevant to IR. An increasing TG level may lead to the elevation of free fatty acids and more extravasation of free fatty acids from fat to non-fat tissue, which may result in IR (13). Furthermore, the insulin sensitivity of skeletal muscle decreases as FPG increases (6, 40, 41). Therefore, the combination of TG and FPG has a high sensitivity for diagnosing IR. As IR promotes endothelial dysfunction, increases the proinflammatory state, and leads to the release of reactive oxygen species, it plays a critical role in the promotion of atherosclerosis progression and plaque formation (42–44).

## Limitations

Several limitations in the current study should be considered. First, this study is a cross-sectional observational study. Although many confounding factors were adjusted, there may be some potential selection bias. Further prospective cohort studies and the inclusion of a control group may provide more precise evidence in future research. Second, the result of 2-h oral glucose tolerance test was not considered in the diagnosis of prediabetes or new-onset T2D, which might limit the sample size that included. Third, the source of the study subjects was a population of asymptomatic, but not healthy, hospitalized patients. Nonetheless, patients with underlying disease and those who received blood glucose-lowering and lipid-lowering therapy were excluded to minimize the effects of the interfering factors. Finally, the study was a single-center study, and multicenter studies may be needed. However, a positive correlation was observed between the TyG index and carotid plaque burden, suggesting that the TyG index may be used as a tool to assess carotid plaque burden in asymptomatic prediabetes and new-onset T2D subjects.

## Conclusions

In conclusion, a high TyG index value was positively associated with a high carotid plaque burden in subjects with prediabetes and new-onset T2D. Clinicians should pay close attention to the TyG index in these populations, as it could help such patients receive the greatest benefit from early intervention.

## Data Availability Statement

The original contributions presented in the study are included in the article/supplementary material, further inquiries can be directed to the corresponding author/s.

## Ethics Statement

The studies involving human participants were reviewed and approved by Academic Ethics Committee of Shaoxing People's Hospital. Written informed consent for participation was not required for this study in accordance with the National Legislation and the Institutional Requirements.

## Author Contributions

Z-zJ was responsible for conceptualization, investigation, and writing the original draft. J-bZ, H-lS, S-sZ, S-qT, and Y-yT contributed to data interpretation and data collection. T-aJ and X-tL designed the study and contributed to critically revising the manuscript. All authors have read and approved the final manuscript.

## Funding

This work was supported by Zhejiang Provincial Natural Science Foundation of China (LQ20H180003), the National Key R&D Program of China (2018YFC0114900), Development Project of National Major Scientific Research Instrument (82027803), National Natural Science Foundation of China (81971623), Key Project of Natural Science Foundation of Zhejiang Province (LZ20H180001), Zhejiang Medicine and Health Science and Technology Project (2021KY1147), and Shaoxing Medical Key Discipline (2019SZD05).

## Conflict of Interest

The authors declare that the research was conducted in the absence of any commercial or financial relationships that could be construed as a potential conflict of interest.

## Publisher's Note

All claims expressed in this article are solely those of the authors and do not necessarily represent those of their affiliated organizations, or those of the publisher, the editors and the reviewers. Any product that may be evaluated in this article, or claim that may be made by its manufacturer, is not guaranteed or endorsed by the publisher.
